# Mediating effect of resilience and fear of COVID-19 on the relationship between social support and post-traumatic stress disorder among campus-quarantined nursing students: a cross-sectional study

**DOI:** 10.1186/s12912-023-01319-4

**Published:** 2023-05-16

**Authors:** Dongmei Zhang, Li Qin, Anle Huang, Congzhi Wang, Ting Yuan, Xiaoping Li, Liu Yang, Jing Li, Yunxiao Lei, Lu Sun, Mingming Liu, Huan Liu, Lin Zhang

**Affiliations:** 1 School of Nursing, Wannan Medical College, 22 Wenchang West Road, Higher Education Park, Wuhu City, An Hui Province P. R. China; 2 The Dean’s Office, Wannan Medical College, 22 Wenchang West Road, Higher Education Park, Wuhu City, An Hui Province P. R. China; 3 Department of Hemodialysis, The First Affiliated Hospital of Wannan Medical College, 2 Zheshan West Road, Wuhu City, An Hui Province P. R. China

**Keywords:** COVID-19, PTSD, Resilience, Fear, Social support, Mediating role

## Abstract

**Background:**

The emergency of Omicron variants, spreading in China and worldwide, has sparked a new wave of the coronavirus disease 2019 (COVID-19) pandemic. The high infectivity and persistence of the pandemic may trigger some degrees of post-traumatic stress disorder (PTSD) for nursing students experiencing indirect trauma exposure to the epidemic, which hinders the role transition from students to qualified nurses and exacerbates the health workforce shortage. Thus, it’s well worth an exploration to understand PTSD and its underlying mechanism. Specifically, PTSD, social support, resilience, and fear of COVID-19 were selected after widely literature review. This study aimed to investigate the relationship between social support and PTSD among nursing students during COVID-19, to address the mediating role of resilience and fear of COVID-19 between social support and PTSD, and to provide practical guidance for nursing students’ psychological intervention.

**Methods:**

From April 26 to April 30, 2022, 966 nursing students from Wannan Medical College were selected by the multistage sampling method to fill the Primary Care PTSD Screen for the fifth edition of the Diagnostic and Statistical Manual of Mental Disorders (DSM-5), Brief Resilience Scale, Fear of COVID-19 Scale, and Oslo 3 Items Social Support Scale. Data were analyzed by descriptive statistics, spearman’s correlation analysis, regression analysis, and path analysis.

**Results:**

15.42% of nursing students had PTSD. There were significant correlations between social support, resilience, fear of COVID-19, and PTSD (*r* =-0.291 ~ 0.353, *P* <0.001). Social support had a direct negative effect on PTSD (β =-0.216; 95% confidence interval, CI: -0.309~-0.117), accounting for 72.48% of the total effect. Analysis of mediating effects revealed that social support influenced PTSD through three indirect pathways: the mediated effect of resilience was statistically significant (β =-0.053; 95% CI: -0.077~-0.031), accounting for 17.79% of the total effect; the mediated effect of fear of COVID-19 was statistically significant (β =-0.016; 95% CI: -0.031~-0.003), accounting for 5.37% of the total effect; the chain mediating effect of resilience and fear of COVID-19 was statistically significant (β =-0.013; 95% CI: -0.022~-0.006), accounting for 4.36% of the total effect.

**Conclusion:**

The social support of nursing students not only directly affects PTSD, but also indirectly affects PTSD through the separate and chain mediating effect of resilience and fear of COVID-19. The compound strategies targeted at boosting perceived social support, fostering resilience, and controlling fear of COVID-19 are warranted for reducing PTSD.

## Background

Since the official announcement from the World Health Organization (WHO) of the coronavirus disease 2019 (COVID-19) pandemic, the sanitary Crisis has swept the world for more than two years and is still from its conclusion [1–3]. The emergency of Omicron variants, currently spreading in China and worldwide, has sparked a new wave of the COVID-19 pandemic [4, 5]. The high infectivity and persistence of the pandemic may trigger various mental issues, such as anxiety, fear, and posttraumatic stress disorder (PTSD) [6, 7]. PTSD is a psychological response to traumatic events, including re-experiencing, avoiding, negative changes in emotions, and hyperarousal [8, 9]. Previous studies [10–12] have shown that both direct and indirect trauma exposure may trigger PTSD. During the COVID-19, healthcare workers, who directly exposed to traumatic events, have been reported a high prevalence of PTSD for treating and caring for COVID-19 patients and witnessing patients suffering and death [13, 14]. College students have shown some degrees of PTSD for experiencing indirect trauma exposure to the epidemic [15, 16]. One study [17] has reported that 44.5% of college nursing students who were home-quarantined and online education had PTSD during the first wave of the pandemic.

The new wave of the COVID-19 pandemic was also a public health emergency that has had an extremely significant impact on the Chinese education system [18], as the government has imposed strict control measures to require college students to lockdown and stay in their dorms to maintain social distancing to prevent the spread of the virus in high-risk areas. Long-term isolation and uncertainty easily worsen their psychological conditions [15, 19]. The COVID-19 and quarantine easily lead to a range of consequences for college students: sudden school confinement, increasing time of access to social media and excessive exposure to COVID-19 news, chronic and acute stress, fear of infection, and worrying about their work and economic future [15, 17, 20, 21]. All of these indirect trauma exposures to the epidemic may trigger PTSD [15, 17, 20]. Compared with adults, adolescents are a more vulnerable group [22].

Due to new cases and variants ebb and flow during COVID-19, the world needs amounts of nurses and demands them to be healthier, more competent, and more motivated than ever. However, high incidences of mental issues among nursing students seem to widen the gap between the increasing demand for high-quality care and the prospective reservoir of qualified nurses. PTSD may be significantly associated with decreased memory and learning abilities, which ultimately lead to poor academic performance in college [22]. Moreover, PTSD is more likely to be a group phenomenon in the context of the pandemic. Therefore, it is crucial for educational administrators and nursing students to place a value on PTSD and its underlying mechanism.

Social support can buffer the negative impacts of main stressors on physical health [23]. The cognitive model of PTSD [24] points out that social support following a traumatic event may impact victims to make sense of the event and prevent PTSD from further development. A meta-analysis [25] has corroborated a correlation between social support and PTSD in young people. Kumpfer’s transactional model of resilience [26] offers a theoretical framework for the present study as follows. External environmental factors such as social support interact to buffer or exacerbate negative impact; stressors or challenges activate the resilience process; with the help of resiliency factors, one person bounces back with resilient reintegration. On the contrary, the individual will be trapped in adversity, leading to maladjustment and a series of psychological and behavioral problems. When the individual experiences a traumatic event, the fear is triggered because of the worldview affected by anxiety. The pandemic nature of COVID-19 has caused higher fears worldwide than ever, and its immediate consequences have given rise to many unprecedented challenges to the education systems [20, 27, 28]. Previous studies [15, 29] have shown that fear is the most significant risk factor of PTSD during COVID-19. Moreover, the etiological theory of PTSD [30] points out that social support as a protective factor affects PTSD by influencing people’s traumatic cognitive assessment, emotional state, and coping strategies. So social support can affect PTSD by influencing fear which is an emotional state following traumatic cognitive assessment. Kumpfer’s transactional model of resilience [26] proposes that social support affects traumatic stress adjustment by influencing resilience. Therefore, when facing the epidemic, students may undergo multiple pressures and feelings such as fear, and some maladjusted students may develop psychological problems such as PTSD, and social support can affect PTSD by affecting individual resilience and fear.

After widely literature review [17, 27, 29, 31–33], there is a lack of research on the relationships between social support, resilience, fear of COVID-19, and PTSD among nursing students, and it was well worth an exploration to understand PTSD and its underlying mechanism. To fill the knowledge gap, our study aimed to investigate the relationship between social support, resilience, fear of COVID-19, and PTSD in nursing students, to provide practical guidance for nursing students’ psychological intervention.

**Fig. 1:**
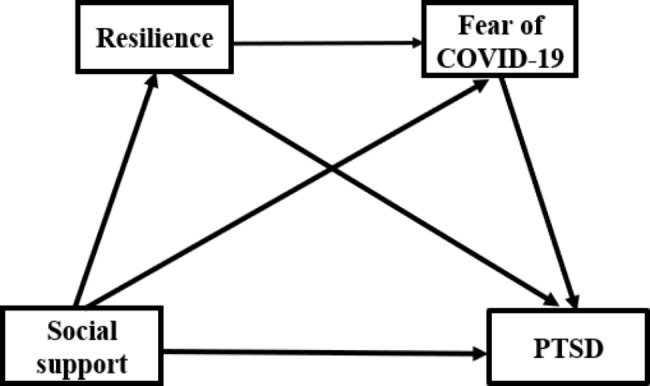
Hypothetical model of the relationships between social support, resilience, fear of COVID-19 and PTSD Note: PTSD: post-traumatic stress disorder; COVID-19: coronavirus disease 2019

Accordingly, we hypothesized as follows: (1) social support is a negative correlation with PTSD. (2) resilience plays a mediating role between social support and PTSD. (3) fear of COVID-19 plays a mediating role between social support and PTSD. (4) resilience and fear of COVID-19 play a chain mediating role between social support and PTSD (Fig. 1).

## Methods

### Participants and procedures

The target population encompassed nursing students from a medical college in Anhui province. According to the criterion suggested by Kendall (10 to 20-fold the number of items and expanded at least 10%) [34], a sample size of no less than 231 was computed since the total items of four scales in this study is 21. Multistage sampling was conducted to select participants. Firstly, we stratified by grade, including 4 grades from freshman to senior. Secondly, 50% of classes (10 ~ 11 classes) in each grade were randomly selected [35]. Thirdly, all students (25 ~ 30 students) in each class were selected by cluster sampling. The inclusion criteria were a full-time nursing student on campus during campus quarantine and consent to participate. Questionnaire star was used to issue the questionnaires. Before data collection, informed consent was obtained from all participants. Once participants agree, they can access the questionnaire-filling interface and click on their choice. Finally, 1008 questionnaires were received from April 26 to April 30, 2022, about one month after the school issued a notice on March 25th, 2022 on the campus quarantine, of which 966 (95.83%) were qualified questionnaires.

### Instrument

#### Sociodemographic information

According to prior studies [6, 15, 29], the sociodemographic variables were collected, including sex, age, academic year, the degree of online classroom adaptation, the stress of COVID-19 on the study, life, and employment.

### The primary care PTSD screen for DSM-5 (PC-PTSD-5)

PC-PTSD-5 was revised by Prins et al. [36] according to the DSM-5 criteria for PTSD. It is a five-item self-report tool. Items on it are scored dichotomously as zero (0 = No) or one (1 = Yes). Cheng et al. [12] translated PC-PTSD-5 into Chinese through the translating-callback method and confirmed the reliability and validity of this scale. The Chinese version has robust psychometric properties, and a cutoff score of two has been recommended in the Chinese version [12, 37]. The Cronbach’s α coefficient of PC-PTSD-5 was 0.830 in Cheng et al. ’s study and 0.841 in our research.

### Fear of COVID-19 scale (FCV-19 S)

FCV-19 S was developed by Ahorsu et al. [38]. It is a seven-item self-reporting measurement tool for evaluating the degree of fear of COVID-19. Its items are rated on a 5-point Likert-type scale. The answers are ranging from 1 (strongly disagree) to 5 (strongly agree). And the highest possible total score on the FCV-19 S is 35, with higher scores indicating greater fear. The Cronbach’s α coefficient of FCV-19 S was 0.820 in the original scale [38]. Feng et al. [39] translated FCV-19 S into Chinese through the Brislin model and corroborated the Chinese version had excellent reliability and validity in Chinese medical students. The Cronbach’s α coefficient of FCV-19 S was 0.924 in Feng et al. ’s study [39] and 0.905 in our study.

### Brief resilience scale (BRS)

BRS was developed by Smith et al. [40]. It is a 6-items self-reporting measurement tool, including 3 positive and 3 negative items. All items are scored on a 5-item Likert-type scale ranging from 1 (strongly disagree) to 5 (strongly agree). The total scores range from 6 to 30, with higher scores indicating better resilience. BRS was a reliable tool. The Cronbach’s α coefficient of BRS was ranging from 0.800 to 0.910 in different samples. The BRS was translated into Chinese and verified using the back-translation procedure [41]. Next, two pilot studies were carried out in the southeast and northwest, and the Chinese version was without any differences in geography and culture. Finally, the reliability and validity of the Chinese version were assessed and confirmed good psychometric properties in undergraduates, and the Cronbach’s α coefficient was 0.71. The reliability and validity of the scale were also verified in Chen et al. ’s study [42]. The Cronbach’s α coefficient was 0.792 in the present study.

### Oslo 3-item Social Support scale (OSS-3)

OSS-3 was applied in measuring the level of social support of nursing students. Despite consisting of only three items as a self-reporting assessment tool, OSS-3 has good psychometric properties and covers different areas of perceived social support such as the sense of concern from other people [43]. And it has been used for several large-scale population-based surveys [44, 45]. A score ranging from 3 to 8, 9 to 11, and 12 to 14 are classified as poor, moderate, and strong social support, respectively. The Cronbach’s α coefficient of OSS-3 was 0.640 in Kocalevent et al.’s study [43]. Feng et al. [46] adapted OSS-3 to Chinese culture and proved good reliability and validity. The Cronbach’s α coefficient of OSS-3 was 0.821 in Feng et al. ’s study [46] and 0.687 in this research.

### Data analysis

The SPSS 25.0 was employed to implement descriptive statistics and correlation analysis with *P* < 0.05 (two-tailed test) as statically significant. The chi-square test was used to analyze if the students’ PTSD has any differences based on sex and academic year. Spearman’s correlation analysis was employed to measure the correlations among social support, resilience, fear of COVID-19, and PTSD. The model 6 of SPSS PROCESS macro, a 5000-sample Bootstrap procedure, and 95% confidence interval (CI) were used for regression analysis, where the effect of sociodemographic correlates (sex, academic year, academic pressure, life stress, and job-hunting stress) was adjusted for [47]. The AMOS 23.0 was adopted to conduct a path analysis for testing the prior hypothesis, and the model was assessed by multiple fit indicators [48]. Moreover, standardized coefficients of direct and indirect effects were estimated with 95% CI according to the bias-corrected confidence intervals method with 5000 Bootstrap samples [47, 48].

## Ethical considerations

The study was approved by the ethical committee of the College of Nursing of Wannan Medical College (20,220,004), and all participating students provided informed consent before the data collection.

## Results

### Participants’ characteristics

The average age was 20.42±1.51 years among the 966 nursing students. As shown in Table 1, there were 284 (30.33%) freshmen, 207 (21.43%) sophomores, 277 (28.67%) juniors, and 198 (19.57%) Senior. In terms of job-hunting stress, 464 (48.03%) nursing students were in a state of high stress. In terms of academic pressure, 377 (39.03%) nursing students were under high pressure. In terms of life stress, 335 (34.68%) nursing students suffered from high pressure. Moreover, 9.01% of nursing students were not adaptable to online classes. Of the 966 nursing students, 149 were screened out PTSD, with a prevalence of 15.42%. As shown in Table 2, respondents with high levels of PTSD were found among the seniors who would graduate from their school two months later, significantly higher than freshmen and juniors (*χ*^2^ = 9.972, *P* = 0.019).

**Table 1:** Background characteristics of nursing students (*n* = 966)

Variables		*N* (%)
Sex	Male	221 (22.88)
	Female	745 (77.12)
Academic year	Freshman	293 (30.33)
	Sophomore	207 (21.43)
	Junior	277 (28.67)
	Senior	189 (19.57)
Online classroom adaptation	Inadaptable	87 (9.01)
	Moderate	396 (40.99)
	Adaptable	483 (50.00)
Academic pressure	Low	150 (15.53)
	Moderate	439 (45.44)
	High	377 (39.03)
Life stress	Low	160 (16.56)
	Moderate	471 (48.76)
	High	335 (34.68)
Job-hunting stress	Low	154 (15.94)
	Moderate	348 (36.03)
	High	464 (48.03)

**Table 2:** Demographic characteristics of PTSD (*n* = 966)

Variables		PTSD		*χ* ^2^	*P*
		No	Yes		
Sex	Male	179 (21.91%)	42 (28.19%)	2.815	0.093
	Female	638 (79.09%)	107 (71.81%)		
Academic year	Freshman	257 (31.46%)	36 (24.16%)	9.972	0.019
	Sophomore	173 (21.18%)	34 (22.82%)		
	Junior	240 (29.38%)	37 (24.83%)		
	Senior	147 (17.99%)	42 (28.19%)		

### Relations of variables

PTSD was significantly correlated with social support (*r* =-0.246, *P* < 0.01), resilience (*r* =-0.281, *P* < 0.01), and fear of COVID-19 (*r* = 0.253, *P* < 0.01). Besides, social support was obviously correlated with resilience (*r* = 0.353, *P* < 0.01) and fear of COVID-19 (*r* =-0.205, *P* < 0.01). Moreover, resilience was obviously correlated with fear of COVID-19 (*r* = -0.291, *P* < 0.01).

### Harman’s univariate analysis

In the present study, multicollinearity has been not found as the variance explained by the first factor was 25.95%, which was less than 40% [35].

### Regression analysis

Social support positively predicted resilience (β = 0.494, *P* <0.001), and negatively predicted the fear of COVID-19 (β =-0.242, *P* <0.05) and PTSD (β =-0.085, *P* <0.001). Resilience negatively predicted fear of COVID-19 (*β* =-0.394, *P* <0.001) and PTSD (β =-0.062, *P* <0.001). Fear of COVID-19 positively predicted PTSD (β = 0.033, *P* <0.001) (Table 3).

**Table 3:** Results of regression analysis from PROCESS macro testing

Variables	Resilience			Fear of COVID-19			PTSD		
	*Se*	*β*	*t*	*Se*	*β*	*t*	*Se*	*β*	*t*
Sex	0.235	-0.719	-3.066^**^	0.408	0.511	1.251	0.089	-0.205	-2.311^*^
Academic year	0.085	0.173	2.030^*^	0.155	-0.070	-0.447	0.034	0.071	2.103^*^
Academic pressure	0.157	-0.407	-2.594^*^	0.272	0.303	1.114	0.057	-0.022	-0.377
Job-hunting stress	0.151	0.011	0.070	0.271	-0.134	-0.494	0.060	-0.030	-0.508
Life stress	0.162	-0.368	-2.266^*^	0.262	0.900	3.431^**^	0.056	0.082	1.469
Social support	0.044	0.494	11.170^***^	0.100	-0.242	-2.413^*^	0.021	-0.085	-4.032^***^
Resilience				0.063	-0.394	-6.237^***^	0.013	-0.062	-4.793 ^***^
Fear of COVID-19							0.009	0.033	3.853^***^
*F*		26.183			13.910			15.380	
*P*		< 0.001			< 0.001			< 0.001	
*R* ^*2*^		0.150			0.108			0.128	

^*^P <0.05, ^**^P <0.01, ^***^P <0.001

### Mediator analysis

The goodness-of-fit of the mediating model was satisfactory with chi-square/degree of freedom (CMIN/DF) = 0.790, goodness of fit index (GFI) = 0.998, adjusted goodness of fit index (AGFI) = 0.994, normed fit index (NFI) = 0.994, Tucker-Lewis index (TLI) = 1.000, incremental fit index (IFI) = 1.000, comparative fit index (CFI) = 1.000, root-mean-square error of approximation (RMSEA) <0.001 (Table 4) [48].

**Table 4:** Evaluation the goodness-of-fit of the mediating model

Model	CMIN/DF	GFI	AGFI	NFI	TLI	IFI	CFI	RMSEA
Mediating model	0.790	0.998	0.994	0.994	1.000	1.000	1.000	< 0.001
Standard value	< 5.000	> 0.900	> 0.900	> 0.900	> 0.900	> 0.900	> 0.900	< 0.08

**Fig. 2:**
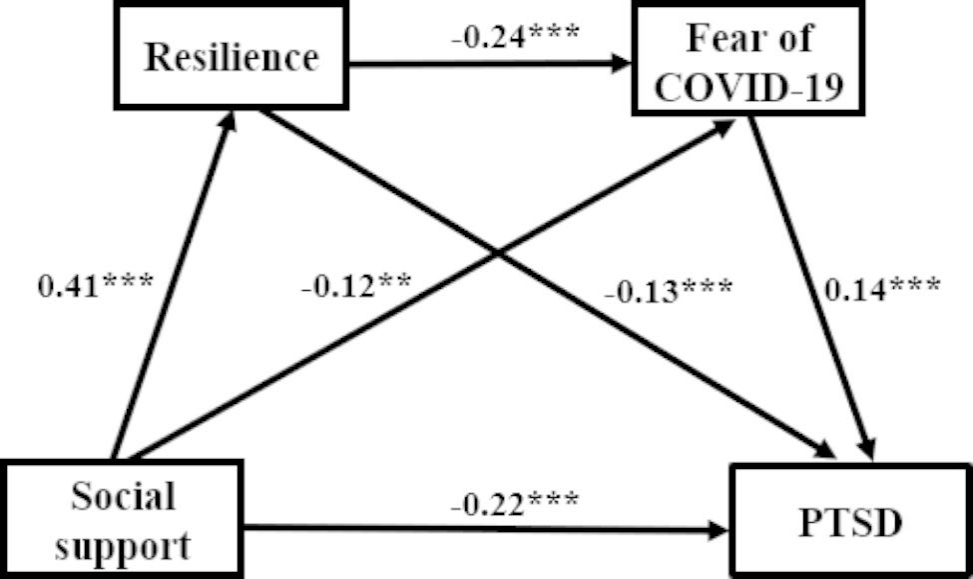
Path model showing resilience and fear of COVID-19 mediating the relationship between social support and PTSD Note: ^**^P<0.01; ^***^P<0.001; PTSD: post-traumatic stress disorder; COVID-19: coronavirus disease 2019

As presented in Table 5; Fig. 2, social support had a direct negative effect on PTSD (β =-0.216; 95% CI: -0.309~-0.117), which confirmed and supported the hypothesis1, accounting for 72.48%% of the total effect. Analysis of mediating effects revealed that social support influenced PTSD through three indirect pathways: the mediated effect of resilience was statistically significant (β = -0.053; 95% CI: -0.077~-0.031), accounting for 17.79% of the total effect; the mediated effect of fear of COVID-19 was statistically significant (β =-0.016; 95% CI: -0.031~-0.003), accounting for 5.37% of the total effect; the chain mediating effect of resilience and fear of COVID-19 was statistically significant (β =-0.013; 95% CI: -0.022~-0.006), accounting for 4.36% of the total effect. The results of indirect effects confirmed and supported the hypothesis 2, 3, and 4 respectively.

**Table 5:** Effects estimate of the hypothesized model (standardized coefficients)

	Structural paths	Effect value	SE	95% CI	%
Direct effects	Social support→PTSD	-0.216	0.049	-0.309 ~ -0.117	72.48%
Indirect effects	Social support→Resilience→PTSD	-0.053	0.012	-0.077 ~ -0.031	17.79%
	Social support→Fear of COVID-19→PTSD	-0.016	0.007	-0.031 ~ -0.003	5.37%
	Social support→Resilience→Fear of COVID-19→PTSD	-0.013	0.004	-0.022 ~ -0.006	4.36%
Total indirect effects		-0.082	0.014	-0.109 ~ -0.055	27.52%
Total effects		-0.298	0.040	-0.374 ~ -0.215	100.00%

## Discussion

As nursing students are the main reserve force against COVID-19, it is especially important to understand PTSD and its underlying mechanism and give timely psychological intervention. This research examined PTSD among nursing students and the relationship among social support, resilience, fear of COVID-19 and PTSD during the new wave pandemic and lockdown. In general, our research confirmed the previous hypotheses, and the results will provide a theoretical basis for interventions to improve nursing students’ mental health.

### Characteristics of PTSD in nursing undergraduates

This study found as high as 48.03% of nursing students with high job-hunting stress, 39.03% with high academic pressure, and 34.68% with high life stress. This study also showed that 15.42% of the sampled students were screened out PTSD, which was a little higher than the college students reported by Liao et al. [16], and approximately compared to the youth in China following the first wave pandemic in 2020 [6, 49]. Obviously, confronted with manifold pressures and challenges from employment, studying, life, excessive exposure to COVID-19 news, and lockdown, the youth are apt to be troubled by PTSD. Accordingly, it is necessary to emphasize the prevention, screening, and psychological intervention of PTSD among adolescent students during the pandemic.

Further study found that seniors had significantly higher levels of PTSD than freshmen and juniors, in accordance with a precious study on university students [29]. When facing the variants ebb and flow and medical new cases during COVID-19, senior nursing students are concerned about their careers ahead and further study. However, many areas strictly restricted entry and many medical-related employers postponed recruitment in the golden period of job hunting during the epidemic lockdown period. And the uncertainty of their career ahead led to high employment pressure. Moreover, although senior nursing students have been at the end of the internship and were not necessary to participate in online learning during the pandemic lockdown, they were facing high learning pressure such as graduation thesis defense, graduation examination, and postgraduate reexamination. Additionally, they also experience constant stress such as fear of infection and separation from family, which increased the risk of triggering PTSD [14, 29]. Therefore, psychological counseling and spiritual comfort for senior nursing students should be placed great emphasis on. In addition, schools and employers should also take corresponding measures, such as expanding employment channels and adopting online recruitment. And schools should provide the required equipment and environment for the online interview and online reexamination of the senior during the quarantine.

### Social support had a direct negative effect on PTSD

This study showed that social support had a direct negative effect on PTSD, which was in accordance with previous studies [50, 51]. Social support is real or available social resources in case of need [30]. It includes four aspects such as emotional support, instrumental support, informational support, and appraisal support [52]. So, people with high levels of social support would have a relatively big possibility to re-engage with lives following the traumatic event and may offer an outlet for people to pour out their hearts and troubles [53], all of which are associated with reducing PTSD.

### Resilience and fear of COVID-19 played mediating roles between social support and PTSD among nursing undergraduates respectively

In addition, our study proved that resilience and fear of COVID-19 mediated the relationship between social support and PTSD respectively, and the mediating effect of resilience was greater than fear of COVID-19. The positive effect of social support on PTSD may be enhanced by resilience and attenuated by fear of COVID-19.

Resilience embodies the individual’s qualities that enable one to adapt and thrive faced with adversity and be regarded as a measure of stress coping ability [54, 55]. Kumpfer’s transactional model of resilience [26] suggests that person-environment interactional processes are self-integration which is necessary for change, and benefit to resilient reintegration as positive life outcomes. Therefore, social support, as a protective factor focusing on stress and coping processes, can help people to bounce back with positive resilient reintegration. Among medical students, resilience was described as several positive qualities, including the ability to take advantage of support systems, self-efficacy, learning from difficulties, persistence, and so forth [56], from which many positive outcomes may stem, including psychological health, good attitude, rationally facing the impact of the epidemic, alleviating the COVID-19 anxiety, improving quality of life and avoiding the occurrence of PTSD on nursing students [31].

During the lockdown of our university, the epidemic has lasted for more than two years for new cases and variants ebb and flow, and completely effective control measures have not been found [57]. Because of the contagious nature of the novel coronavirus and the long lockdown, fear in nursing students was to be triggered easily on this particular occasion. Fear is an adaptive action that elicits defensive behaviors. However, PTSD can be evoked when fear is inappropriately adjusted [58]. And the previous study has demonstrated that fear is a significant predictor of PTSD [29]. According to the pressure buffer theory, emotional support and informational support can prevent PTSD by reducing fear. On the one hand, emotional support and instrumental support can improve one person’s sense of security and belonging, which in turn could reduce an individual’s arousal and distress. On the other hand, instrumental support contributed to lessening the burdens caused by the pandemic directly, reducing the degree of their fear, and thus the mental impacts of the stressor. Therefore, social support that decreased the degree of fear and instilled resilience in nursing students could be a benefit for preventing PTSD during the pandemic.

### Resilience and fear of COVID-19 played a chain mediating effect between social support and PTSD

We also proved that resilience and fear of COVID-19 played a chain mediating effect between social support and PTSD, following the related theory that social support needs to mediate the process to buffer the stress by trauma [59]. Therefore, social support would reduce the occurrence of PTSD by improving the psychological levels of individuals. Social support can improve the sense of control over stressful events and alleviate negative emotions by providing support of emotion, instruments, and information to avoid the occurrence of PTSD.

In the present study, social support reduced the risk of PTSD by influencing resilience and fear of COVID-19. It was obvious that social support played a key role, particularly for individuals facing negative emotions [60]. Social support has been testified to help to improve resilience [52]. Individuals with high resilience are more apt to adopt positive coping strategies and pour out their hearts to others [61]. And the act of pouring their heart out may play a sedative effect which is indispensable for an individual’s mental health [62–64]. support of emotion, instrument, and information reduced the stress on nursing students from learning, life, and job-hunting, and are benefit for improving their resilience. Moreover, most of the nursing students have mastered certain methods of emotion regulation as they have finished the psychology courses, which helped them to maintain or quickly recover a good mentality during the lockdown. However, the higher resilience they have, the more optimistic and confident, and stronger ability to recover. Resources such as well social support, optimism, positive coping, and the range of those evaluated by previous resilience measures were benefit for improving the ability to recover from stress. And there may be a direct relationship between the ability to recover and health outcomes [40]. When facing the lockdown, they would not feel fear as they take positive adjustments. Information-processing theories of PTSD [65] suggest that the fear network in memory causes individuals to develop symptoms of PTSD such as hypervigilant, intrusion, and avoidance. Therefore, social support can influence the PTSD of nursing students through the chain mediating effect of resilience and COVID-19 fear.

### Limitations

In this study, there are several limitations. Firstly, the cross-sectional study limited the causality among social support, resilience, COVID-19 fear, and PTSD, so a longitudinal study should be carried out to test these results. Secondly, the respondents were nursing students in one school. Therefore, further work is needed to enlarge the sample coverage. However, this study examined PTSD among nursing students and its influencing mechanism during the new wave pandemic and campus quarantine, and the results may offer the theoretical foundation for interventions to improve nursing students’ mental health.

## Conclusion

The mental health of nursing students is directly related to the prospective reservoir of qualified nurses, and high-quality care in the future. Rightfully so, it deserves greater attention to strengthen the mental health of nursing students. The nursing students are apt to be troubled by PTSD during campus quarantine. The social support of nursing students not only directly affects PTSD, but also indirectly affects PTSD through the separate and chain mediating effect of resilience and fear of COVID-19. Therefore, it is necessary to focus on the prevention, screening, and psychological intervention of PTSD among nursing students. Accordingly, the compound strategies targeted at boosting social support, fostering resilience, and controlling fear of COVID-19 are warranted for reducing PTSD. In terms of schools, some measures should be taken to relieve the stress among nursing students, such as colorful online activities and online and offline psychological counseling during the campus quarantine. Moreover, the teachers may take full advantage of medical science, integrating the knowledge of coronavirus and the anti-epidemic spirit of healthcare workers into the teaching of professional courses, so as to guide nursing students correctly understand the COVID-19 pandemic, and relieve negative emotions such as fear of COVID-19, improve their professional responsibility as medical students. In the long run, schools should incorporate the improvement of students’ resilience into their talent training plans, optimize nursing curricula, and offer courses related to mental health. Parents should provide students with more emotional support and appropriate material support, and focus on defusing negative emotions. Besides, it is crucial to assist nursing students to regard support networks as resources and to evaluate and make full use of available support resources.

## Data Availability

The datasets generated and/or analyzed during the present study are not publicly available to preserve the anonymity of the participants but are available from the corresponding author at reasonable request.

## Abbreviations

PTSD: Post-traumatic stress disorder
COVID-19: Coronavirus disease 2019
DSM-5: The fifth edition of the Diagnostic and Statistical Manual of Mental Disorders
WHO: World Health Organization
PC-PTSD-5: The Primary Care PTSD Screen for DSM-5
FCV-19S: Fear of COVID-19 Scale
BRS: Brief Resilience Scale
OSS-3: Oslo 3 items social support scale
CI: Confidence interval
CMIN/DF: Chi-square/degree of freedom
GFI: Goodness of fit index
AGFI: Adjusted goodness of fit index
NFI: Normed fit index
TLI: Tucker-Lewis index
IFI: Incremental fit index
CFI: Comparative fit index
RMSEA: Root-mean-square error of approximation

## References

[1] Thakur V, Ratho RK (2022). OMICRON (B.1.1.529): a new SARS-CoV-2 variant of concern mounting worldwide fear. J Med Virol.

[2] Kandeel M, Mohamed MEM, Abd El-Lateef HM, Venugopala KN, El-Beltagi HS (2022). Omicron variant genome evolution and phylogenetics. J Med Virol.

[3] Mohapatra RK, Sarangi AK, Kandi V, Azam M, Tiwari R, Dhama K (2022). Omicron (B.1.1.529 variant of SARS-CoV-2); an emerging threat: current global scenario. J Med Virol.

[4] Viana R, Moyo S, Amoako DG, Tegally H, Scheepers C, Althaus CL (2022). Rapid epidemic expansion of the SARS-CoV-2 Omicron variant in southern Africa. Nature.

[5] Saxena SK, Kumar S, Ansari S, Paweska JT, Maurya VK, Tripathi AK (2022). Characterization of the novel SARS-CoV-2 Omicron (B.1.1.529) variant of concern and its global perspective. J Med Virol.

[6] Liang L, Ren H, Cao R, Hu Y, Qin Z, Li C (2020). The Effect of COVID-19 on Youth Mental Health. Psychiatr Q.

[7] Bao Y, Sun Y, Meng S, Shi J, Lu L (2020). 2019-nCoV epidemic: address mental health care to empower society. Lancet.

[8] North CS, Surís AM, Smith RP, King RV (2016). The evolution of PTSD criteria across editions of DSM. Ann Clin Psychiatry.

[9] Prestia AS (2022). PTSD and the COVID-19 Continuum. Nurse Lead.

[10] Hassan M, Jordan F, Tawfick W (2022). Mental stress in health care professionals during COVID-19 outbreak. Ir J Med Sci.

[11] Yin Q, Sun Z, Liu T, Ni X, Deng X, Jia Y (2020). Posttraumatic stress symptoms of health care workers during the corona virus disease 2019. Clin Psychol Psychother.

[12] Cheng P, Jasinski N, Zheng W, Yadava A, Wang L, Li L (2021). Psychometric Properties of the primary care PTSD screen for DSM-5: findings from Family Members of Chinese Healthcare Workers during the outbreak of COVID-19. Front Psychiatry.

[13] Lai J, Ma S, Wang Y, Cai Z, Hu J, Wei N (2020). Factors Associated with Mental Health Outcomes among Health Care Workers exposed to Coronavirus Disease 2019. JAMA Netw Open.

[14] Kar N, Kar B, Kar S (2021). Stress and coping during COVID-19 pandemic: result of an online survey. Psychiatry Res.

[15] Khan AH, Sultana MS, Hossain S, Hasan MT, Ahmed HU, Sikder MT (2020). The impact of COVID-19 pandemic on mental health & wellbeing among home-quarantined bangladeshi students: a cross-sectional pilot study. J Affect Disord.

[16] Liao W, Luo X, Ye Z (2022). Relationship between college students’ social support and post-traumatic stress symptoms in a pandemic: chained multiple mediating effects of sense of control and avoidace coping strategies. Chin J Health Psychol.

[17] Gao J, Wang F, Guo S, Hu F (2021). Mental Health of nursing students amid Coronavirus Disease 2019 Pandemic. Front Psychol.

[18] Li Y, Fan W, Wang W, Han J, Pan J, Bao H (2022). A school cluster outbreak of COVID-19 caused by SARS-CoV-2 Omicron variant. Chin J Public Health.

[19] Rubin GJ, Wessely S (2020). The psychological effects of quarantining a city. BMJ.

[20] Brooks SK, Webster RK, Smith LE, Woodland L, Wessely S, Greenberg N (2020). The psychological impact of quarantine and how to reduce it: rapid review of the evidence. Lancet.

[21] Shimizu K (2020). 2019-nCoV, fake news, and racism. Lancet.

[22] Tian Y, Zhou X, Wu X (2018). The relationship between traumatic exposure and substance abuse among adolescents after the Wenchuan Earthquake: the mediating role of PTSD and attachment. J Psychol Sci.

[23] Thoits PA (2011). Mechanisms linking social ties and support to physical and mental health. J Health Soc Behav.

[24] Murray H, Grey N, Wild J, Warnock-Parkes E, Kerr A, Clark DM (2020). Cognitive therapy for post-traumatic stress disorder following critical illness and intensive care unit admission. Cogn Behav Therap.

[25] Allen L, Jones C, Fox A, Copello A, Jones N, Meiser-Stedman R (2021). The correlation between social support and post-traumatic stress disorder in children and adolescents: a meta-analysis. J Affect Disord.

[26] Kumpfer KL, Summerhays JF (2006). Prevention approaches to enhance resilience among high-risk youth: comments on the papers of Dishion & Connell and Greenberg. Ann N Y Acad Sci.

[27] Nguyen HT, Do BN, Pham KM, Kim GB, Dam HTB, Nguyen TT (2020). Fear of COVID-19 Scale-Associations of its scores with health literacy and health-related behaviors among medical students. Int J Environ Res Public Health.

[28] Knipe D, Evans H, Marchant A, Gunnell D, John A (2020). Mapping population mental health concerns related to COVID-19 and the consequences of physical distancing: a Google trends analysis. Wellcome Open Res.

[29] Tang W, Hu T, Hu B, Jin C, Wang G, Xie C (2020). Prevalence and correlates of PTSD and depressive symptoms one month after the outbreak of the COVID-19 epidemic in a sample of home-quarantined chinese university students. J Affect Disord.

[30] Wang Y, Chung MC, Wang N, Yu X, Kenardy J (2021). Social support and posttraumatic stress disorder: a meta-analysis of longitudinal studies. Clin Psychol Rev.

[31] Berdida DJE, Grande RAN (2022). Academic stress, COVID-19 anxiety, and quality of life among nursing students: the mediating role of resilience. Int Nurs Rev.

[32] Joseph RA, Turner T, Lee C, Akers SW, Whorley E, Goodrich C (2022). Impact of COVID-19 on nursing students: factors Associated with PTSD Risk. J Christ Nurs.

[33] Cobo-Cuenca AI, Fernández-Fernández B, Carmona-Torres JM, Pozuelo-Carrascosa DP, Laredo-Aguilera JA, Romero-Gómez B (2022). Longitudinal study of the Mental Health, Resilience, and post-traumatic stress of senior nursing students to nursing graduates during the COVID-19 pandemic. Int J Environ Res Public Health.

[34] JQ F (2002). Advanced Medical Statistics.

[35] Fang D, Gao Y, Dai B, Zhou M, Li J (2011). Application of different statistical sampling methods in prescription evaluation. China Pharm.

[36] Prins A, Bovin MJ, Smolenski DJ, Marx BP, Kimerling R, Jenkins-Guarnieri MA (2016). The primary care PTSD screen for DSM-5 (PC-PTSD-5): development and evaluation within a veteran primary care sample. J Gen Intern Med.

[37] Huang RW, Shen T, Ge LM, Cao L, Luo JF, Wu SY (2021). Psychometric Properties of the Chinese Version of the primary care post-traumatic stress disorder Screen-5 for medical staff exposed to the COVID-19 pandemic. Psychol Res Behav Manag.

[38] Ahorsu DK, Lin CY, Imani V, Saffari M, Griffiths MD, Pakpour AH (2022). The fear of COVID-19 scale: development and initial validation. Int J Ment Health Addict.

[39] Feng Q, Huang C, Jia Y, Liu T, Jia H, Wang K (2021). Reliability and validity of the chinese version of fear of coronavirus disease 2019 scale. Acad J Second Military Med Univ.

[40] Smith BW, Dalen J, Wiggins K, Tooley E, Christopher P, Bernard J (2008). The brief resilience scale: assessing the ability to bounce back. Int J Behav Med.

[41] Fung SF (2020). Validity of the brief resilience scale and brief resilient coping scale in a chinese sample. Int J Environ Res Public Health.

[42] Chen W, Liu J, Luo J, Liu GQ (2020). Reliability and validity of the chinese version of brief resilience scale. Chin J Clin Psychol.

[43] Kocalevent RD, Berg L, Beutel ME, Hinz A, Zenger M, Härter M, Nater U, Brähler E (2018). Social support in the general population: standardization of the Oslo social support scale (OSSS-3). BMC Psychol.

[44] Alemu D, Soboka M, Tesfaye E, Ahmed G, Tesfaye Y (2020). Alcohol Use Disorder and Associated factors among Jimma University undergraduate students. Psychol Res Behav Manag.

[45] Dalgard OS, Dowrick C, Lehtinen V, Vazquez-Barquero JL, Casey P, Wilkinson G (2006). Negative life events, social support and gender difference in depression: a multinational community survey with data from the ODIN study. Soc Psychiatry Psychiatr Epidemiol.

[46] Feng ZP. Exploring the utilization and influencing factors of Health Services for Chronic Disease Patients based on Andersen behavioral model. Dalian Medical University; 2019.

[47] Zhang S, Tang Y, Yong S (2022). The influence of gratitude on pre-service teachers’ career goal self-efficacy: chained intermediary analysis of meaning in life and career calling. Front Psychol.

[48] Wu ML (2010). Structural equation modeling.

[49] Cao C, Wang L, Fang R, Liu P, Bi Y, Luo S (2022). Anxiety, depression, and PTSD symptoms among high school students in china in response to the COVID-19 pandemic and lockdown. J Affect Disord.

[50] Vig KD, Mason JE, Carleton RN, Asmundson GJG, Anderson GS, Groll D (2020). Mental health and social support among public safety personnel. Occup Med (Lond).

[51] Jung H, Jung SY, Lee MH, Kim MS (2020). Assessing the Presence of post-traumatic stress and turnover intention among nurses Post-Middle East Respiratory Syndrome Outbreak: the importance of Supervisor Support. Workplace Health Saf.

[52] Casapulla S, Rodriguez J, Nandyal S, Chavan B (2020). Toward resilience: medical students’ perception of Social Support. J Am Osteopath Assoc.

[53] Cohen S (2004). Social relationships and health. Am Psychol.

[54] Connor KM, Davidson JR (2003). Development of a new resilience scale: the Connor-Davidson Resilience Scale (CD-RISC). Depress Anxiety.

[55] Morse JM, Kent-Marvick J, Barry LA, Harvey J, Okang EN, Rudd EA (2021). Developing the Resilience Framework for nursing and Healthcare. Glob Qual Nurs Res.

[56] Howe A, Smajdor A, Stöckl A (2012). Towards an understanding of resilience and its relevance to medical training. Med Educ.

[57] Kumar S, Thambiraja TS, Karuppanan K, Subramaniam G (2022). Omicron and Delta variant of SARS-CoV-2: a comparative computational study of spike protein. J Med Virol.

[58] Brewin CR (2001). A cognitive neuroscience account of posttraumatic stress disorder and its treatment. Behav Res Ther.

[59] Coyne JC, Downey G (1991). Social factors and psychopathology: stress, social support, and coping processes. Annu Rev Psychol.

[60] Hendrickson ZM, Kim J, Tol WA, Shrestha A, Kafle HM, Luitel NP (2018). Resilience among Nepali Widows after the death of a spouse: “That was my past and now I have to see my Present. Qual Health Res.

[61] Sexton MB, Byrd MR, von Kluge S (2010). Measuring resilience in women experiencing infertility using the CD-RISC: examining infertility-related stress, general distress, and coping styles. J Psychiatr Res.

[62] Wang A, Bai X, Lou T, Pang J, Tang S (2020). Mitigating distress and promoting positive aspects of caring in caregivers of children and adolescents with schizophrenia: mediation effects of resilience, hope, and social support. Int J Ment Health Nurs.

[63] Yu NX, Chan CL, Zhang J, Stewart SM (2016). Resilience and vulnerability: prolonged grief in the bereaved spouses of marital partners who died of AIDS. AIDS Care.

[64] Ong HL, Vaingankar JA, Abdin E, Sambasivam R, Fauziana R, Tan ME (2018). Resilience and burden in caregivers of older adults: moderating and mediating effects of perceived social support. BMC Psychiatry.

[65] Foa EB, Riggs DS, Massie ED. The impact of fear activation and anger on the efficacy of exposure treatment for posttraumatic stress disorder. Behav Ther. 1995;26:487–99.

